# Structural and Mechanical Characteristics of Cu_50_Zr_43_Al_7_ Bulk Metallic Glass Fabricated by Selective Laser Melting

**DOI:** 10.3390/ma12050775

**Published:** 2019-03-06

**Authors:** Xiaoyang Lu, Mussokulov Nursulton, Yulei Du, Wenhe Liao

**Affiliations:** 1 School of Mechanical Engineering, Nanjing University of Science and Technology, Nanjing 210094, China; njust_luxiaoyang@163.com (X.L.); ac_milan_barsa@mail.ru (M.N.); 2 Luoyang Ship Material Research Institute, Luoyang 471023, China

**Keywords:** bulk metallic glasses, selective laser melting, Cu_50_Zr_43_Al_7_, mechanical properties

## Abstract

In this work, the structural and mechanical characteristics of Cu_50_Zr_43_Al_7_ bulk metallic glass (BMG) fabricated by selective laser melting (SLM) are studied and the impacts from the SLM process are clarified. Cu_50_Zr_43_Al_7_ alloy specimens were manufactured by the SLM method from corresponding gas-atomized amorphous powders. The as-built specimens were examined in terms of phase structure, morphologies, thermal properties and mechanical behavior. The x-ray diffraction and differential scanning calorimetry results showed that structural relaxation and partial crystallization co-exist in the as-fabricated Cu_50_Zr_43_Al_7_ glassy samples. The nano- and micro- hardness and the elastic modulus of the SLM-fabricated Cu_50_Zr_43_Al_7_ BMG were higher than CuZrAl ternary BMGs with similar compositions prepared by conventional mold casting, which can be attributed to the structural relaxation in the former sample. However, the macro compressive strength of the SLM-fabricated Cu_50_Zr_43_Al_7_ BMG was only 1044 MPa mainly due to its porosity. This work suggests that the SLM process induced changes in structural and mechanical properties are significant and cannot be neglected in the fabrication of BMGs.

## 1. Introduction

Bulk metallic glasses (BMGs) are promising structural materials because of their unique mechanical, physical and chemical properties [1,2,3]. However, due to their limited ability to form glass, it is difficult to fabricate BMGs with large cross sections or complex shapes by traditional mold casting methods. To overcome such size and shape restrictions, powder metallurgy methods, such as hot-pressing sintering and spark plasma sintering techniques, have been adopted to prepare BMGs [4,5]. However, the consolidation of amorphous powders into bulk samples by these methods requires high pressure and molds. So, the manufacturing of large BMGs with complex shapes remains a problem. Selective laser melting (SLM), a laser additive manufacturing technique, can obtain a cooling rate as high as 10^3^–10^4^ K/s during the point-by-point and layer-by-layer forming period. It can thus realize the freeform fabrication of metallic materials and has recently been adopted in various attempts to produce BMGs [6]. To date, some Fe- [7,8,9], Zr- [10,11,12], Al- [13,14] and Ti-based [15] glass–forming alloys have been fabricated by SLM, preliminarily verifying its capabilities in producing BMGs in intricate shapes and large sizes. It is known that Cu-based BMGs usually possess a higher strength, better ductility and are relatively lower in cost [16,17]. However, the application of SLM and infrared lasering in the manufacturing Cu-based BMGs is restricted by the high thermal conductivity and the high reflectivity of Cu-based alloys, resulting in greater heat loss and an inadequate melting of the powders during the SLM process.

This work seeks to uncover the structural and mechanical characteristics of SLM-fabricated Cu-based BMGs and sort out the related impact of SLM processing. The Cu_50_Zr_43_Al_7_ BMG, a typical ternary Cu-based bulk glass–forming alloy, was chosen for the study and fabricated by SLM. Its structural and mechanical characteristics were analyzed.

## 2. Materials and Methods

**Figure 1:**
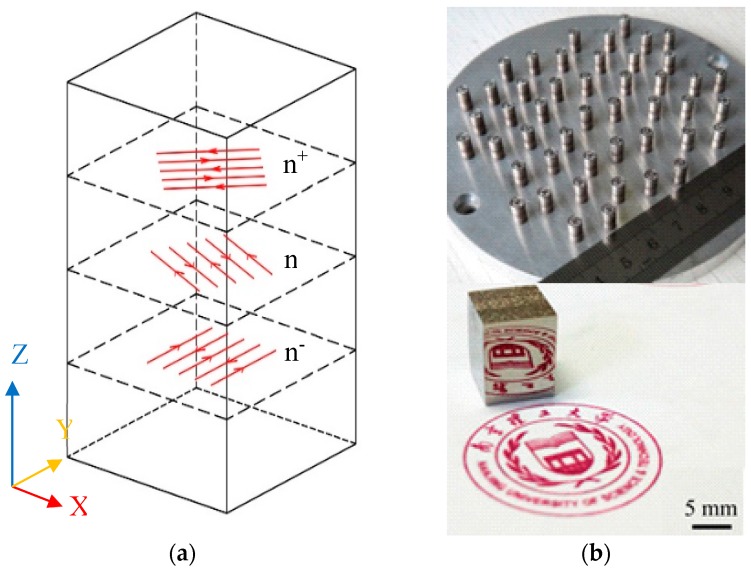
(**a**) Scanning strategy of the present selective laser melting; (**b**) The morphology and length of the SLM-fabricated specimens.

Cu_50_Zr_43_Al_7_ powders were prepared by argon gas atomization. The as-atomized powders with a particle size between 10 and 50 μm were selected for SLM experiments. Cu_50_Zr_43_Al_7_ cylindrical and cubic samples were fabricated using a 3D printer (YLM-120 SLM, manufactured by Jiangsu Yongnian Tech. Co. Ltd., Suzhou, China). As listed in Table 1, the SLM fabrication parameters for these cylinders were as follows: the laser power *P* = 150 and 190 W; the scanning speed *v* = 2000, 2200, and 2400 mm/s; the hatch distance *h* = 0.1 and 0.12 mm; the layer thickness *d* = 0.03 mm; and the laser energy density *E* was in the range of 20.8–31.7 J/mm^3^ calculated using the formula *E*= *P*/(*h* × *v* × *d*) (J/mm^3^). The SLM fabrication process was performed under an argon atmosphere with an oxygen content of below 100 ppm. The rotation angle of the laser scanning direction of adjacent layers was 67° as shown in Figure 1a. The size of the SLM-fabricated cylinder samples was 4 mm in diameter and 8 mm in height. The samples for each experimental condition were fabricated for several repetitions at a time (as shown in Figure 1b). 

The amorphous nature of the as-prepared Cu_50_Zr_43_Al_7_ powders and SLM-fabricated samples was verified by X-ray diffraction (XRD, D8 Advance, Bruker-AXS, Karlsruhe, Germany) with Cu Kα radiation and differential scanning calorimetry (DSC). The transmission electron microscopy (TEM) observations were performed on a JEM-200CX instrument (JEOL, Tokyo, Japan). Specimens for the TEM observation were prepared by standard twin-jet electrolytic thinning with an HNO_3_–CH_3_OH electrolyte (volume ratio 7:3). Uniaxial compression tests were conducted with an Instron-8801 (Instron, Norwood, America) testing machine at room temperature at an engineering strain rate of 1 × 10^−4^ s^−1^, and more than three SLM-fabricated BMG compression specimens with diameter of 4 mm and height of 8 mm were used. The compression fracture morphologies of the SLM samples were investigated by scanning electron microscopy (SEM, Quanta 250FEG, FEI, Hillsboro, America). Standard Berkovich nanoindentation tests were conducted at room temperature using a CSM™ Nanoindenter (NHT, CSM, Peseux, Switzerland). The experiments were run at a constant loading rate of 100 mN/min with a peak load of 50 mN. The density of the SLM-fabricated samples was determined by the Archimedes method. Vickers hardness experiments were also taken with a peak load of 0.5 N.

## 3. Results and Discussion

**Figure 2:**
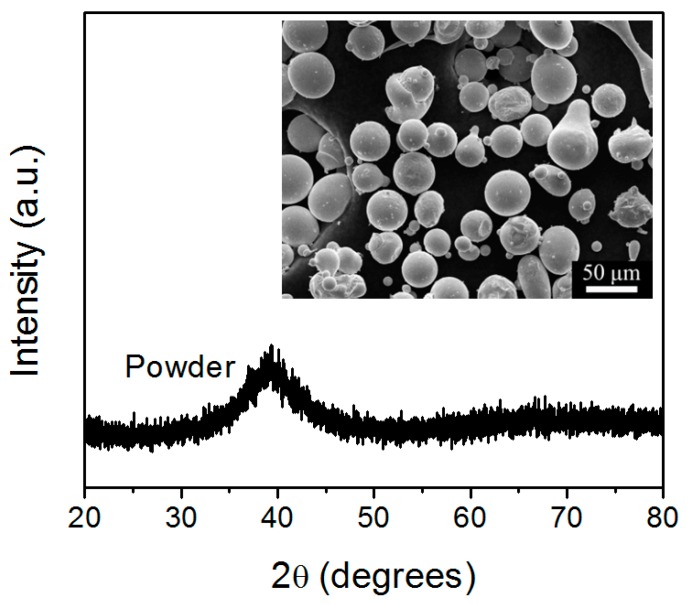
X-ray diffraction (XRD) pattern of the as-atomized Cu_50_Zr_43_Al_7_ powders. The inset depicts a scanning electron microscopy (SEM) image of the powders.

Figure 2 shows the XRD pattern of the as-atomized Cu_50_Zr_43_Al_7_ powders. The pattern exhibits a characteristic broad diffraction without any detectable crystalline Bragg peaks, indicating that fully amorphous structures were obtained for the Cu_50_Zr_43_Al_7_ particles with dimensions smaller than 50 μm. Obviously, the cooling rate during the gas atomization process was sufficient to suppress the formation of the crystalline phases. As shown in the inset of Figure 2, most of the as-atomized Cu_50_Zr_43_Al_7_ powders exhibited spherical or near spherical shape with a smooth surface, which was conducive to the smooth running of the SLM process

**Figure 3:**
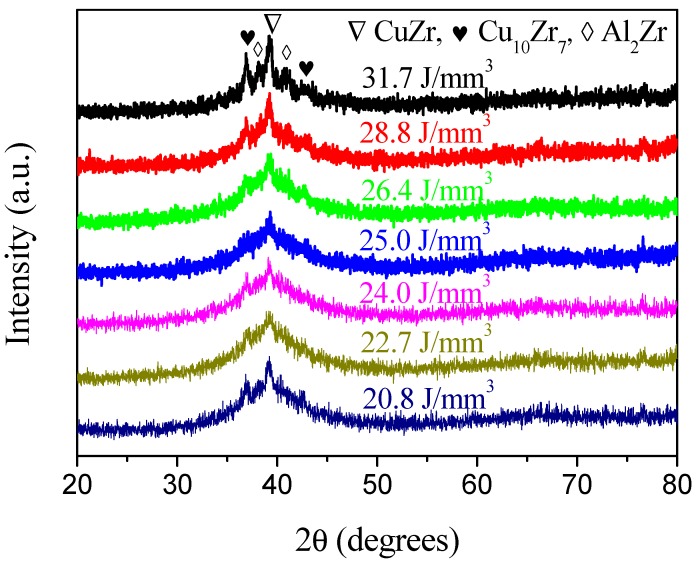
XRD patterns of the SLM-fabricated Cu_50_Zr_43_Al_7_ specimens under different laser energy densities.

**Figure 4:**
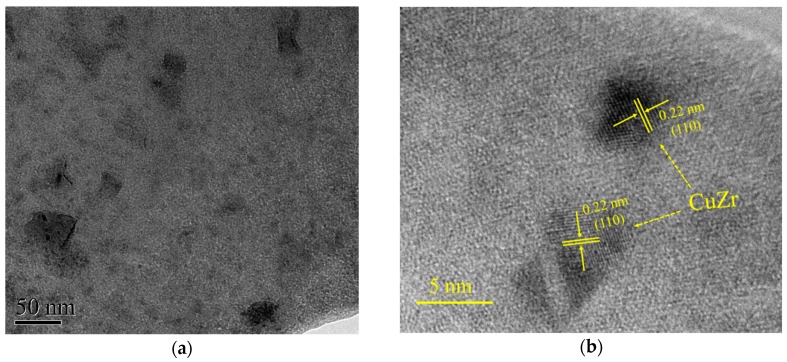
(**a**) Transmission electron microscopy (TEM) morphology and (**b**) high resolution transmission electron microscopy (HRTEM) image of SLM-fabricated Cu_50_Zr_43_Al_7_ bulk metallic glasses (BMG) under a laser energy density of 25 J/mm3.

In the present SLM process, the laser energy density was varied in order to study its influence on the amorphization of the SLM-fabricated Cu_50_Zr_43_Al_7_ samples. As can be seen from Figure 1b, when the applied laser energy density was in the range of 20.8–31.7 J/mm^3^, all the samples were successfully produced by SLM. A cubic sample with a polished lateral surface was also shown in Figure 1b. As can be seen, a mirror-like surface can be obtained by polishing, indicating the high quality of the SLM-fabricated Cu-based BMG. No obvious cracking or layer delamination was observed by visual inspection of the exterior surface. Figure 3 shows the XRD patterns taken on the cross sections of the SLM-fabricated samples under different laser energy densities. It can be seen that for the samples fabricated with the laser energy density of 31.7, 28.8 and 26.4 J/mm^3^, some crystalline phases occurred within the amorphous matrix, which can be attributed to crystallization at heat affect zones under relatively high laser energy. Based on previous reports [18,19], these crystalline phases can be identified as CuZr, Cu_10_Zr_7_ and Al_2_Zr phases, respectively. Interestingly, for the samples fabricated under a low laser energy density of 20.8, 22.7 and 24 J/mm^3^, obvious crystalline phases could also be detected, which might have been due to the crystallization of the un-melted powders under relatively low laser energy. For the samples fabricated with a laser energy density of 25 J/mm^3^, a nearly fully amorphous structure was obtained. However, a poor superimposed peak on the main amorphous maximum indicated that slight crystallization was inevitable in the SLM-fabricated BMG samples. In order to further investigate the crystalline phases in the SLM-fabricated Cu-based BMG sample, TEM observations were performed. Figure 4 shows the TEM images of the SLM-fabricated Cu_50_Zr_43_Al_7_ BMG under a laser energy density of 25 J/mm^3^. As can be seen from Figure 4a, many crystalline phases dispersed in the amorphous matrix. These crystalline phases were irregular in shape and their size ranged from several to dozens of nanometers. Figure 4b shows the enlarged HRTEM (high resolution transmission electron microscopy) image of the SLM-fabricated Cu-based BMG. It is evident that the nano-sized crystalline phases precipitated from the glassy matrix. As indicated in the figure, the crystalline phases were identified as CuZr phase, consistent with the XRD result. The samples SLM-fabricated under a laser energy density of 25 J/mm^3^ were used in the following studies, because of their nearly fully amorphous structure.

**Figure 5:**
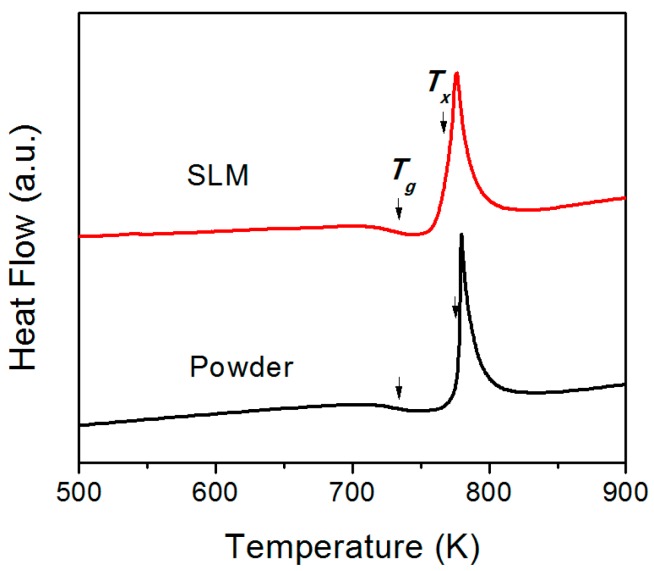
Differential scanning calorimetry (DSC) curves of as-atomized Cu_50_Zr_43_Al_7_ metallic glass powders and the SLM-fabricated Cu_50_Zr_43_Al_7_ BMG sample.

Figure 5 shows the DSC curves of as-atomized Cu_50_Zr_43_Al_7_ powders and an SLM-fabricated sample with a laser energy density of 25 J/mm^3^. Both of them showed the typical features of the amorphous phase. The values of glass transition temperature (*T_g_*) and relaxation enthalpy (*ΔH_r_*) were 733.1 K, 1.3 J/g and 733.4 K, 1.9 J/g, respectively for the as-atomized Cu_50_Zr_43_Al_7_ powders and the SLM-fabricated BMG samples. Apparently, the higher relaxation enthalpy of the SLM-fabricated BMG samples shows that they experienced structural relaxation during the SLM process. The existence of structural relaxation in the SLM-fabricated sample can be also proved by the fact that the values of the onset temperature of crystallization (*T_x_*, 777.7 K) for the as-atomized metallic glass powders were significantly higher than those (768.3 K) for the SLM-fabricated BMG samples. As reported previously [20], structural relaxation may lead to a decrease in the *T_x_* of BMGs. In addition, the values of the crystallization enthalpy (*ΔH_c_*, 49.6 J/g) for the as-atomized Cu_50_Zr_43_Al_7_ powders were significantly higher than those (*ΔH_c_*, 40.2 J/g) for the SLM-fabricated BMG sample, which indicates that partial crystallization occurred in the latter sample. SLM is a layer by layer deposition process, during which there exists a fusion zone in the current layer, a remelting zone and a heat-affected zone in the former solidified layers. The specific heat transfer status in different zones led to the occurrence of structural relaxation and partial crystallization in the SLM-fabricated BMGs. The present work implies that structural relaxation and partial crystallization co-exist in the SLM-fabricated BMGs.

**Figure 6:**
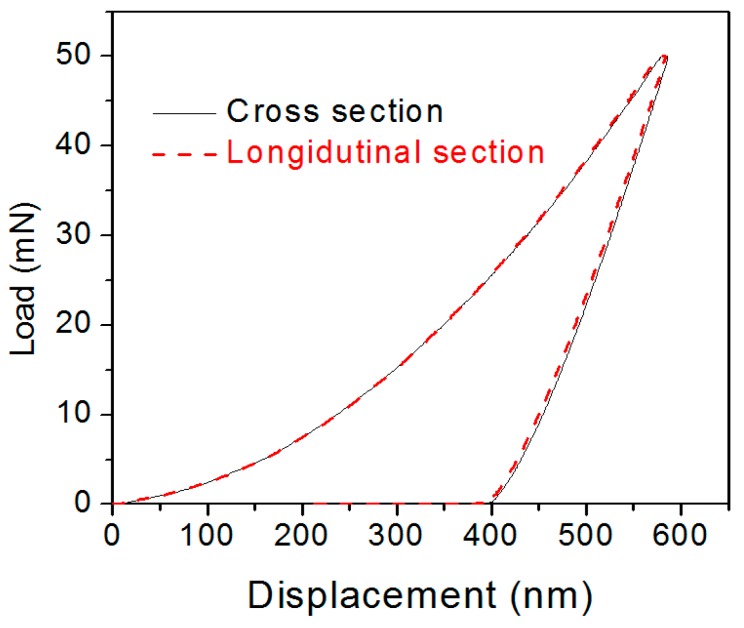
The fitted load-displacement (P-h) curves for nanoindentation on the cross and longitudinal sections of SLM-fabricated Cu_50_Zr_43_Al_7_ BMG samples.

**Figure 7:**
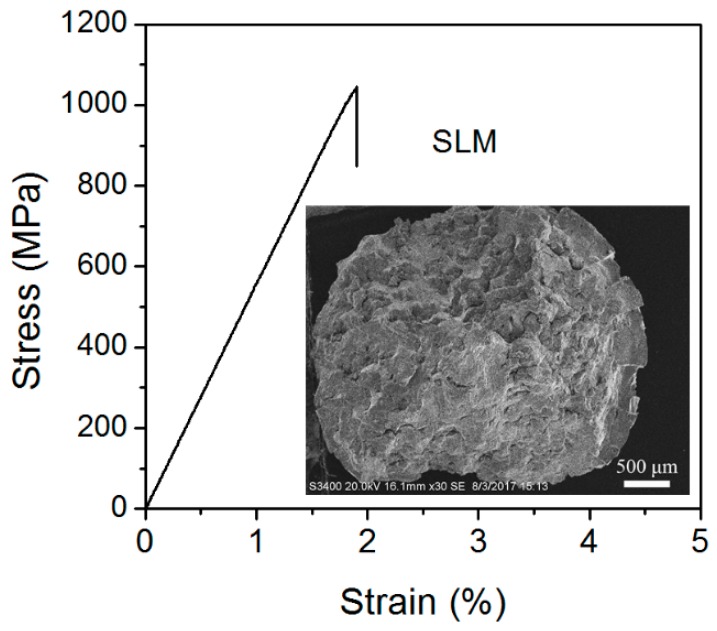
The stress–strain curve in the compression of SLM-fabricated Cu_50_Zr_43_Al_7_ BMG rod samples. The inset depicts an SEM image of the fractured surface.

Figure 6 shows the nanoindentation load-displacement (P-h) curves measured on the cross and longitudinal sections of the SLM-fabricated Cu_50_Zr_43_Al_7_ BMG cubic samples with a side length of 10 mm. Following the methods proposed by Oliver and Pharr [20], the nanoindentation hardness (H_IT_/MPa) and elastic modulus (E_IT_) values were calculated from the load-displacement curves. The results are listed in Table 2, together with the microscopic Vickers hardness and those from the cast Cu-based BMGs with similar compositions in the literature [22,23]. As can be seen, the hardness and elastic modulus of the SLM-fabricated Cu_50_Zr_43_Al_7_ BMGs were much higher than those of the CuZrAl ternary BMGs prepared by conventional mold casting. This enhancement can mainly be attributed to structural relaxation. As reported previously [24,25], the annealing induced structural relaxation in BMGs resulting from free volume annihilation and atomic rearrangement, hindered the formation and propagation of shear bands. The hardness and elastic modulus of the relaxed BMGs were thus higher than that of the as-cast BMGs. It should be noted that the present SLM-fabricated BMG sample contained crystalline phases embedded in an amorphous matrix, as shown in Figure 3. In the previous work [26], it was revealed that the crystalline phases were softer than the amorphous matrix, thus, it is reasonable to conclude that the increased hardness in the present SLM-fabricated BMG sample was mainly due to the structural relaxation during the SLM process. Figure 7 shows the compressive engineering stress–strain curve of the SLM-fabricated Cu_50_Zr_43_Al_7_ BMG rods. The compressive strength was 1044 MPa, lower than that of the as-cast Cu-based BMGs in previous reports [16,17]. The decrease in compressive strength was also found in other SLM-fabricated BMGs [15]. Obviously, the SLM-fabricated BMGs exhibited higher nanohardness and lower macro compressive strength compared to the corresponding BMGs prepared by traditional casting methods. It is worthy to reveal the reason for this phenomenon. Since SLM is a powder bed fusion additive manufacturing process, the SLM-fabricated samples are usually not completely dense and have many structural defects [7,8,9,10,11,15]. It is known that the macro–mechanical properties of metallic materials are strongly affected by the presence of structural defects. The relative density of the SLM-fabricated Cu_50_Zr_43_Al_7_ BMG sample under a laser energy density of 25 J/mm^3^ was measured to be 0.97 by the Archimedes method. It is known that the relative density can be increased by increasing the laser energy density in the SLM process, however, in our case, a higher laser energy density than 25 J/mm^3^ will lead to the obvious crystallization of the CuZrAl BMG, as shown in Figure 3.

**Figure 8:**
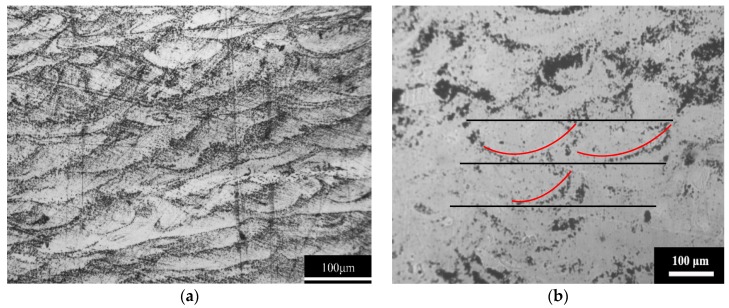
Morphology of solidified molten pools in different zones on the uncorroded section parallel to the build direction: (**a**) zones with densely packed layers; (**b**) zones with a number of pores.

It is known that the SLM-fabricated component is formed by overlapping multi-track and multi-layer molten pools [27]. Thus, the macro–mechanical properties of SLM-fabricated component will be strongly affected by the solidified molten pools. Figure 8a,b show the morphology of the solidified molten pools in different zones on the uncorroded section parallel to the build direction of the as-fabricated Cu_50_Zr_43_Al_7_ BMG. The arc-shape of the molten pool is due to the Gaussian energy distribution of the laser beam, and can be clearly seen in Figure 8a,b. In addition to the densely packed layers, a number of pores were found in the solidified molten pool boundaries of the local zones on the SLM-fabricated BMG sample, which may be the main reason for the low relative density of the SLM-fabricated Cu_50_Zr_43_Al_7_ BMG. Apparently, formation of pores was much easier in the SLM-fabricated BMGs than in the SLM-produced crystalline metallic materials. This can be attributed to the highly viscous nature of the BMG melts [28,29,30]. Compared with the SLM-produced crystalline metallic materials, the BMGs are multicomponent alloys with large atomic size mismatches and a composition near deep eutectic. They show high viscosities that are several orders of magnitude higher than pure metal and common alloy melts [31]. The high viscosity of BMG melts causes difficulties in the spread of the melts, leading to the formation of many pores in the SLM-fabricated BMGs. The presence of pores leads to a decrease of the macro compressive strength of the SLM-fabricated BMGs.

**Figure 9:**
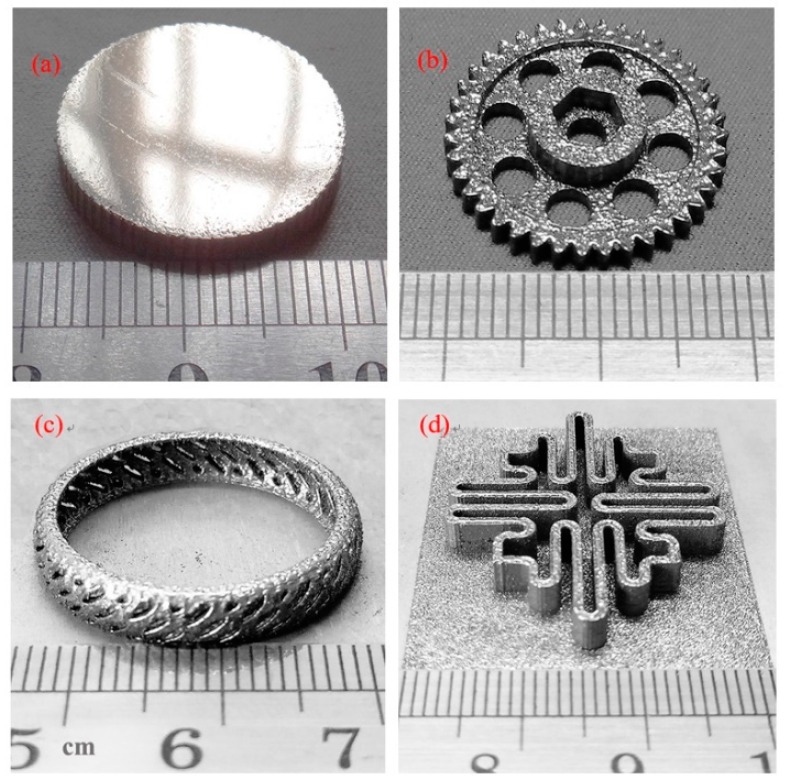
Some large and/or complex glassy Cu_50_Zr_43_Al_7_ parts produced by SLM: (**a**) a disk with diameter of 20 mm; (**b**) a micro gear; (**c**) a ring with hollowed-out structure; (**d**) a part with labyrinth-like structure.

In our work, some Cu_50_Zr_43_Al_7_ BMG specimens with complex and delicate geometries were also fabricated by SLM, as shown in Figure 9, in order to evaluate the processability of the present as-atomized BMG powders. It can be seen that all the specimens have a relatively smooth surface without any macroscopic defects, which indicates that Cu_50_Zr_43_Al_7_ BMG specimens can be well manufactured by the SLM method. The present work implied that SLM is a promising processing avenue for fabricating BMGs with large sizes and complex shapes that may be impossible to obtain by traditional casting methods.

## 4. Conclusions

In conclusion, mainly amorphous Cu_50_Zr_43_Al_7_ BMG specimens are fabricated by SLM. It was found that many nano-sized crystalline phases precipitated from the glassy matrix during the SLM process. The as-prepared BMG specimens without major external cracks had a number of micro-scale pores in the solidified molten pool boundaries due to the high viscosity of the BMG melts, which may be the main reason for the low relative density and high porosity of the SLM-fabricated Cu_50_Zr_43_Al_7_ BMG. The hardness and elastic modulus of the SLM-fabricated Cu_50_Zr_43_Al_7_ BMG was higher than those of the CuZrAl ternary BMGs prepared by conventional mold casting. This can be mainly attributed to structural relaxation during the SLM process. The presence of lots of pores resulted in a decrease in the macro compressive strength of the SLM-fabricated BMGs. Our work proved that SLM is a promising technique for the development of BMGs with complex geometries and large cross sections, and SLM processing brings significant changes to the structural and mechanical properties of BMGs.

## Figures and Tables

**Table 1:** Selective laser melting (SLM) experimental parameters.

*E* (J/mm^3^)	*P* (W)	*v* (mm/s)	*h* (mm)	*d* (mm)
31.7	190	2000	0.10	0.03
28.8	190	2200	0.10	0.03
26.4	190	2400	0.10	0.03
25.0	150	2000	0.10	0.03
24.0	190	2200	0.12	0.03
22.7	150	2200	0.10	0.03
20.8	150	2400	0.10	0.03

**Table 2:** The microscopic Vickers hardness (HV0.5), nanoindentation hardness (H_IT_), elastic modulus (E_IT_) of SLM-fabricated Cu_50_Zr_43_Al_7_ BMG, as well as some casting CuZrAl ternary BMGs.

Sample	Method	HV0.5 (3σ)	H_IT_ (3σ, MPa)	E_IT_ (3σ, GPa)	Ref.
Cu_50_Zr_43_Al_7_ (Cross section)	SLM	550.1 ± 10.9	9313.7 ± 90.6	129.1 ± 3.6	Present
Cu_50_Zr_43_Al_7_ (Longitudinal section)	SLM	555.8 ± 10.6	9415.6 ± 112.5	129.6 ± 4.6	Present
(Cu_50_Zr_50_)_100−x_Al_x_, x = 0~10	as-cast	449.0~541.0	7300.0~8700.0	100.5~117.3	[20]
Cu_46.5_Zr_46.5_Al_7_	as-cast	-	7430.0	118.9	[21]
